# Endoscopic salvage of a dislodged lumen-apposing metal stent in pancreatic pseudocyst drainage

**DOI:** 10.1055/a-2589-1661

**Published:** 2025-05-14

**Authors:** Junfeng Zhang, Fei Li, Jinhua Deng, Tao Zhang

**Affiliations:** 1 Institute of Hepatopancreatobiliary Surgery, Chongqing General Hospital, Chongqing University, Chongqing, China; 2 Chongqing Key Laboratory of Intelligent Medicine Engineering for Hepatopancreatobiliary Diseases, Chongqing, China; 3 Department of Gastroenterology, Yubei District Hospital of Traditional Chinese Medicine, Chongqing, China

**Fig. 1:**
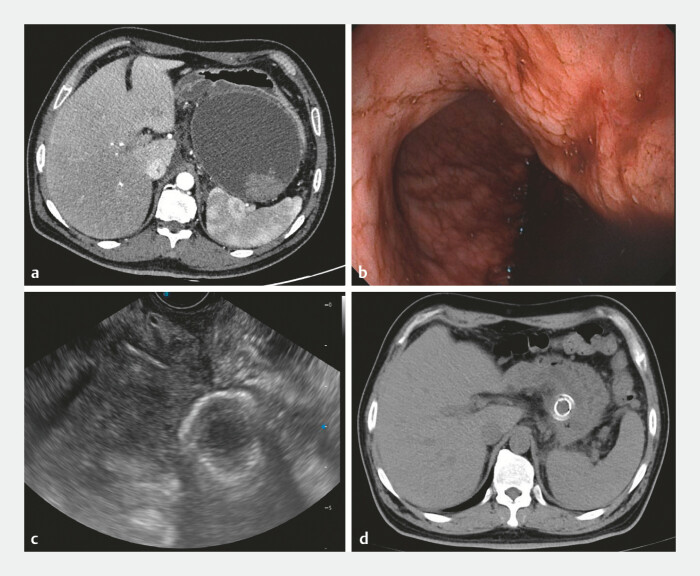
The process of failed LAMS placement and the successful repositioning outcome. **a** Preoperative CT imaging of the pancreatic pseudocyst. **b** First intraoperative puncture showing no LAMS in the gastric cavity. **c** Second intraoperative ultrasound showing the LAMS dislodged into the cyst. **d** Postoperative CT after the second procedure showing successful repositioning of the LAMS and cyst shrinkage. Abbreviation: LAMS, lumen-apposing metal stent.

A 41-year-old man experienced abdominal pain after consuming greasy food and was diagnosed with a pancreatic pseudocyst following acute necrotizing pancreatitis ( Fig. 1 **a** ), undergoing endoscopic ultrasound (EUS)-guided drainage with a lumen-apposing metal stent (LAMS, Boston Scientific). During the cystogastrostomy procedure, the LAMS inadvertently dislodged completely into the pseudocyst ( Fig. 1 **b** ). Double pigtail stents and a nasocystic drainage tube were immediately placed intraoperatively to ensure continuous drainage of the cyst fluid.

One week later, imaging demonstrated significant shrinkage of the pseudocyst ( Fig. 1 **c** ), and the patient remained asymptomatic, with no signs of infection or bleeding. Given the favorable clinical outcome, we decided to attempt endoscopic repositioning of the dislodged LAMS ( Video 1 ). First, the displaced stent was localized within the pseudocyst under EUS guidance. Subsequently, a needle knife was advanced over a guidewire through the original puncture tract, successfully entering the pseudocyst cavity. A 2 cm dilation balloon was then used to expand the tract between the gastric and cyst walls, preparing for the next step.

The process of endoscopically inserting a wire into the cyst, performing dilation, and repositioning the LAMS.Video 1

Using a therapeutic gastroscope (1.0 cm outer diameter) guided by the wire, the operator advanced into the pseudocyst, where extensive necrotic debris was encountered. After repeated careful exploration, the dislodged LAMS was identified. With the assistance of forceps, the position of the LAMS was adjusted. One end of the stent was gently pulled into the gastric lumen, while the other end was kept inside the pseudocyst. The stent was then repositioned to securely anchor between the gastric and cyst walls, successfully re-establishing an effective drainage pathway.

After repositioning, the gastroscope passed smoothly through the LAMS into the pseudocyst, and normal cyst drainage resumed (Fig. 1d). This case demonstrates that endoscopic repositioning of a dislodged LAMS is a feasible and safe strategy, offering an effective and minimally invasive alternative for the treatment of pancreatic pseudocysts.

Endoscopy_UCTN_Code_CPL_1AK_2AG

